# Establishment of epithelial inflammatory injury model using adult kidney organoids

**DOI:** 10.1093/lifemedi/lnae022

**Published:** 2024-05-09

**Authors:** Haoran Du, Liqiang Guo, Jiabei Lian, Huanlu Qiu, Yunuo Mao, Fan Yi, Huili Hu

**Affiliations:** The Key Laboratory of Experimental Teratology, Ministry of Education, Department of Systems Biomedicine, School of Basic Medical Sciences, Shandong University, Jinan 250012, China; Department of Urology, Qilu Hospital, Cheeloo College of Medicine, Shandong University, Jinan 250012, China; The Key Laboratory of Experimental Teratology, Ministry of Education, Department of Systems Biomedicine, School of Basic Medical Sciences, Shandong University, Jinan 250012, China; The Key Laboratory of Experimental Teratology, Ministry of Education, Department of Systems Biomedicine, School of Basic Medical Sciences, Shandong University, Jinan 250012, China; The Key Laboratory of Experimental Teratology, Ministry of Education, Department of Systems Biomedicine, School of Basic Medical Sciences, Shandong University, Jinan 250012, China; The Key Laboratory of Infection and Immunity of Shandong Province, Department of Pharmacology, School of Basic Medical Sciences, Shandong University, Jinan 250012, China; The Key Laboratory of Experimental Teratology, Ministry of Education, Department of Systems Biomedicine, School of Basic Medical Sciences, Shandong University, Jinan 250012, China

**Dear E** **ditor,**

Kidney organoids are emerging as an increasingly applied model system to holds great promise for transplantation to individuals with end-stage renal disease and as a useful tool for kidney research [1, 2]. Several iPSC-derived renal organoids have been established to understand kidney development and disease (e.g. nephritis, renal fibrosis, and polycystic kidney disease, etc.) [3]. However, few disease models originated from adult renal tissue with fully mature cell fates have been set up to recapitulate kidney inflammation or fibrosis [4].

Tumor necrosis factor-alpha (TNFα) is a major proinflammatory and tissue damaged promoting cytokine, which has been implicated in inflammatory renal tissue injury during pathological conditions associated with chronic kidney disease (CKD) or acute kidney injury [5]. Enhanced TNFα production has been observed in renal tubular-related diseases such as cisplatin nephropathy, obstructive nephropathy, and diabetic nephropathy [6]. Evidence in mouse models showed that deletion of TNFα in immune cells exhibited decreased glomerular and tubular injury [7], suggesting its potential role as an adjunct therapeutic strategy. However, the effects of TNFα on renal epithelial cells remained largely unexplored. Here, to mimic TNFα stimulation of proinflammatory events during nephropathy, we treated human normal organoids derived from kidney tissue with TNFα and determined the TNFα-driven changes observed in these organoids.

Human kidney organoids with tubular characters were established from six different donors and maintained through stable passages (passages 4–5) [8]. Following a 24-h treatment with TNFα, a dose-dependent increase in inflammation-related genes, including C-X-C motif chemokine ligand (*CXCL*) 8, interleukin (IL)-6 (*IL-6*) and *IL-1β*, was observed and sustained to 48 h (Fig. 1A). Phase-contrast microscopy revealed increased irregularity in organoid morphology after TNFα stimulation (Fig. 1B). Prolonged exposure to 1000 ng/mL TNFα for 120 h resulted in a reduction in cell viability (Fig. 1C). Moreover, positive signals of γH2AX and LCN2 immunostaining indicated DNA damage after TNFα treatment in organoids (Fig. 1D), suggesting the role of TNFα in promoting inflammation and organoid damage. In addition, the immunostaining of HAVCR1 and MKI67 showed reduced cell proliferation and increased renal damage after TNFα stimulation. (Fig.1E and 1F).

**Figure 1.**
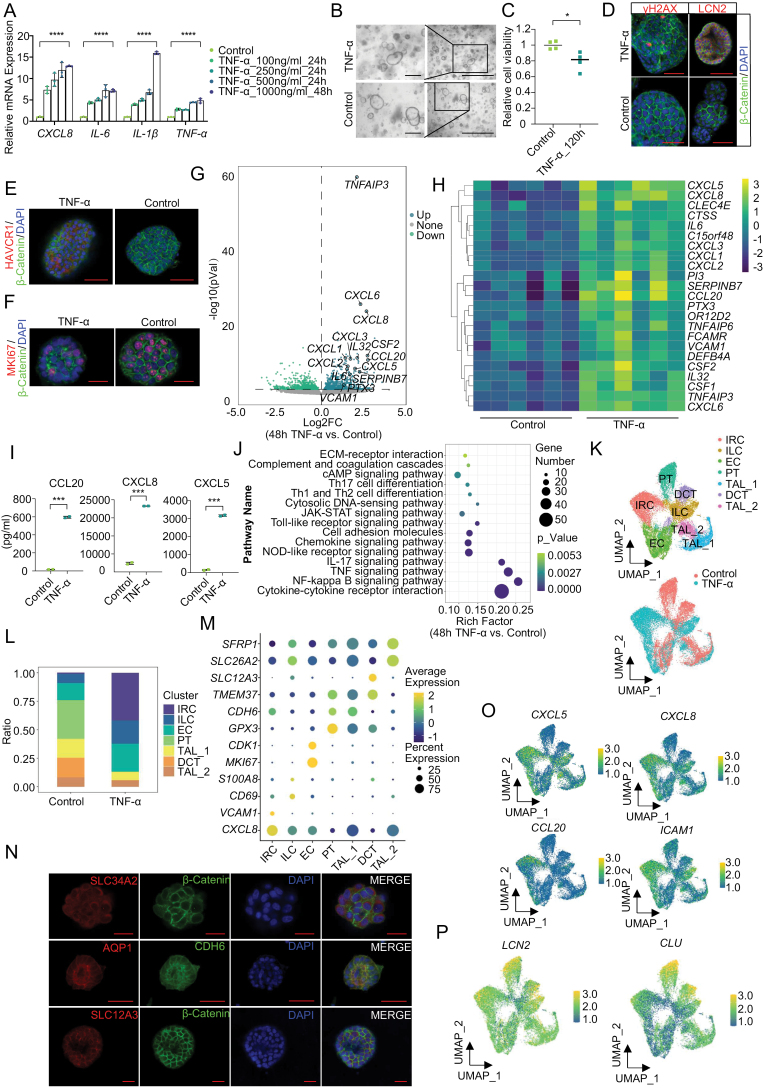
**Establishment of epithelial inflammatory injury model using adult kidney organoids upon TNFα treatment.**(A) The dose-dependent expression of inflammation-related genes in control and organoids treated with TNFα for 24 h or 48 h. Data are represented as mean in triplicate from different donors. (B) Representative images comparing control and organoids treated with 1000 ng/mL TNFα for 48 h. Scale bar = 1000 μm (right), 250 μm (left). (C) The relative cell viability of TNFα-treated organoids (after 120 h) and control organoids. CellTiter-Glo® 3D cell viability assay (Promega, USA) was employed to estimate the cell viability. (D) Immunofluorescent staining of γ-H2AX (left) or LCN2 (right) in control and organoids treated with TNFα. β-Catenin was used to label the cell membrane. Scale bar = 50 μm. (E) Immunofluorescent staining of HAVCR1 and β-Catenin in control and organoids treated with TNFα. Scale bar = 50 μm. (F) Immunofluorescent staining of MKI67 and β-Catenin in control and organoids treated with TNFα. Scale bar = 20 μm. (G) A volcano plot depicting differentially expressed genes in control and organoids treated with TNFα. Light green dots represent genes expressed at lower levels in TNFα-treated organoids while dark green represents genes at higher levels in TNFα treated organoids. *Y*-axis denotes −log_10_*P* values while *X*-axis shows log_2_ fold change values. (H) Heatmap of an inflammatory response gene cluster showing differential expression between six TNFα-treated organoids and their corresponding controls. Expression values for each gene (row) are normalized across all samples (columns) by *Z*-score. Row clustering was applied, and the 23 distinct genes identified by the h-cluster statistic method are shown to illustrate the major expression patterns observed in the data. (I) Secretions of inflammatory chemokines CCL20, CXCL8, and CXCL5 between 48 h after TNFα stimulation and the corresponding control group. Data are represented in duplicate. (J) KEGG analysis of differentially expressed genes between normal organoids and TNFα-treated organoids. The color and size of the dots in the scatterplot represent the range of the negative log_10_-transformed *P*-value and the gene number, respectively. (K) UMAP visualization of nephrogenic cell clusters in kidney organoids, colored by clusters (left) and samples (right). Epithelial cycling cells (EC), proximal tubule (PT), distal convoluted tubule (DCT), inflammatory response cells (IRC), immune-like cells (ILC), thick ascending limb_1 (TAL_1), thick ascending limb_2 (TAL_2). (L) Cell composition distribution for organoids before and after TNFα treatment. (M) Dotplot of major-specific markers for seven cell clusters in kidney organoids. (N) Immunofluorescent staining of SLC34A2 (TAL marker, up, red, scale bar = 20 μm) or AQP1 (PT marker, middle, red, scale bar = 50 μm) or CDH6 (PT marker, middle, green, scale bar = 50 μm) or SLC12A3 (DCT marker, down, red, scale bar = 20 μm) in organoids. β-Catenin (green) was used to label the membrane. (O) UMAP plots representing the expression of inflammatory factors-related markers in kidney organoids including CXCL5, CXCL8, CCL20, and ICAM1. (P) UMAP plots representing the expression of injury-related markers in kidney organoids including LCN2, and CLU.

To comprehensively evaluate the transcriptomic alterations of kidney organoids, we performed RNA sequencing of all six established organoids before or after 48 h of TNFα (1 μg) stimulation (GSE259376). Upon TNFα exposure, the expression of 658 genes was increased, while the expression of 637 genes was decreased in all six organoid lines. TNFα associated inflammatory genes, tumor necrosis factor alpha-induced protein 3 (*TNFAIP3*), *CXCL1*, *CXCL6*, *CXCL8,* C-C motif chemokine ligand 20 (*CCL20*), vascular cell adhesion molecule 1 (*VCAM1*), and *IL6*, were significantly upregulated as shown in Fig. 1G. H-cluster analysis of differentially expressed genes (DEGs) identified a specific cluster consisting of signature inflammatory factors, chemokines, and cytokines that were significantly upregulated after TNFα treatment. The heatmap of the above genes revealed representative molecular changes in the inflammatory response in the epithelial organoids (Fig. 1H). The release of TNFα-induced inflammatory factors was subsequently detected based on the ELISA assay. Notably, the secretion of TNFα-induced inflammatory chemokines, including CCL20, CXCL8, and CXCL5 was indeed significantly increased after 48 h of TNFα stimulation compared with the corresponding control group. (Fig. 1I) (*P* < 0.001). Consequently, exposure to TNFα elicited a cascade of inflammatory responses in organoids such as upregulation of mRNA levels and increased secretion of chemokines. To further comprehensively investigate the molecular changes, we also performed a KEGG analysis on DEGs. Besides the ‘TNF signaling pathway’, inflammation-associated pathways induced by TNFα including ‘cytokine-cytokine receptor interaction’, ‘NF-kappa B signaling pathway’, and ‘chemokine signaling pathway’ were obviously activated (Fig. 1J). In summary, *in vitro,* TNFα-treated kidney organoids create a novel model that faithfully represents the cellular states of the inflammatory response during the progression of renal disease such as nephritis.

To understand the exact cell fates responsible for the inflammatory signatures upon TNFα stimulation, we next sought to delineate the cellular composition of organoids at the single-cell level (data available in GSE259381). A total of 17,760 cells were sequenced and compared between the TNFα-treated and control groups. Six main cell types in both samples were identified including epithelial cycling cells (ECs, MKI67^+^), inflammatory response cells (IRCs, CCL2^+^), proximal tubule (PT, GPX3^+^), distal convoluted tubule (DCT, SLC12A3^+^), thick ascending limb (TAL, SLC26A2^+^), and immune like cells (ILCs, CD69^+^) (Fig. 1K). The cell proportion showed the emergence of distinct major populations of IRCs and ILCs uniquely in TNFα-treated organoids with activated inflammatory factor-related genes and immune cell surface markers as molecular signatures respectively. CD69, a marker of the immune population that responds quickly to chronic inflammation in kidney disease, was significantly upregulated in ILCs. This remark indicates the acquisition of immune-like characteristics by some epithelial cells, supporting the discovery of emerging populations upon injury-induced response. In contrast, PT and DCT clusters, as important cellular components in normal tubular organoids, significantly reduced after TNFα stimulation (Fig. 1L). This is consistent with previously reported reductions in healthy tubular cells following injury [9]. Dotplot depicted the presence of diverse identifiable feature genes based on the published single-cell RNA sequencing (scRNA-seq) data (Fig. 1M) and their expression of functional genes within kidney organoids was confirmed by staining (Fig. 1N). Notably, our customized IRC cluster significantly expressed inflammatory chemokines such as CXCL5, CXCL8, CCL20, and ICAM1 (Fig. 1O). Moreover, TNFα induced injury-associated markers such as *LCN2* and *CLU* were also significantly increased (Fig. 1P). It is speculated that this injury leads to the remarkable decrease of PT and DCT cells, suggesting the effect of TNFα in promoting tubular damage. In summary, our results revealed the successful establishment of an epithelial inflammatory injury model using TNFα-treated kidney organoids.

Elderly patients take a higher risk of CKD progression and failure of tissue repair, although the underlying mechanisms are not fully understood [10]. Next, we detected the differences in sensitivity to inflammatory stimuli between young and old donors using our established TNFα-treated organoid model. Secreted CXCL5 and CXCL8 levels were elevated in renal organoids from both young (35y, 41y) and old (65y, 71y) donors, with higher fold changes in younger organoids (Fig. 2A and 2B). Furthermore, we performed Venn analysis on DEGs from bulk RNA-seq in Fig. 1 to compare the age-related changes in the young (three donors with the age of 34y, 35y, and 41y) and old (three donors with the age of 64y, 65y, and 71y) group. As shown in Fig. 2C and 2D, a conservative increase in inflammatory signals and a decrease in metabolic pathways were detected based on a gene list of 251 overlapping genes altered in both groups. Notably, 732 genes were significantly changed specifically in the younger group while only 240 differentially expressed genes were found in the older group, suggesting a more sensitive response against TNFα exposure in the younger group. The heatmap based on average gene expression of three donors from young or old groups with or without TNFα treatment showed typical genes classified into four groups (Fig. 2E). Group 1 represented inflammation-related genes such as *IL*s, *VCAM1,* and *ICAM1* that were upregulated in both groups. The gene expression profiles of groups 2 and 4 had a trend to decrease or increase specifically in the young group and become comparable with the old group upon TNFα stimulation, indicating an aging-like phenotype induced by TNFα. Also, there are some genes in group 3 like fibronectin were only downregulated in old group. The addition of fibronectin recombinant protein during TNFα treatment significantly increased the proliferation capacity of organoids in old donors (Fig. 2F).

**Figure 2.**
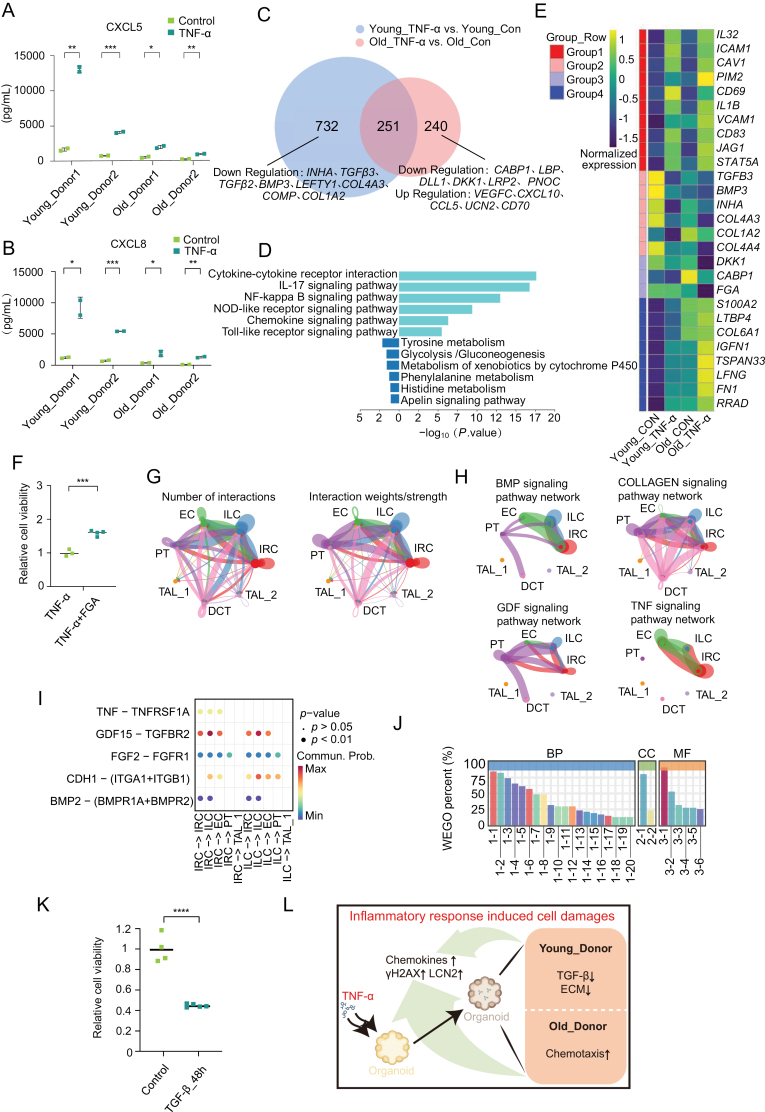
**Donor age is associated with TNFα-induced inflammatory response in organoids.**(A) CXCL5 secretions in two independent groups of young (34y, 41y) and old donor (65y, 71y) derived organoids stimulated with or without TNFα for 48 h. Data are represented in duplicate. (B) CXCL8 secretions in two independent groups of young (34y, 41y) and old donor (65y, 71y) derived organoids stimulated with or without TNFα for 48 h. Data are represented in duplicate. (C) A Venn plot illustrating the intersection of differentially expressed genes (|log_2_FC| ≥ 0) between young donor-derived organoids treated or untreated with TNFα versus older donors. (D) KEGG analysis of 251 conserved differential expressed genes in (C). (E) Heatmap of customized gene groups in organoids from young (34y, 35y, 41y) and old donors (64y, 65y, 71y) with or without TNFα treatment. Expression values for each gene are normalized across all samples. (F) The relative cell viability of TNFα-treated organoids with or without FGA treatment. (G) Circle plots showing the interactions and strengths among different cell types in TNFα treated organoids. Circle sizes represent the number of cells in each cell type. The thickness of each line connecting the cell cluster represents the strength of the communication. (H) Circle plots displaying the inferred network of the ‘TNF’, ‘GDF’，‘BMP’, and ‘COLLAGEN’ signaling pathways among different cell types in TNFα treated organoids. Circle sizes represent the number of cells in each cell type. The thickness of each line connecting the cell cluster represents the strength of the communication. (I) Dot plot showed communication probabilities in TNFα, FGF, CDH1, BMP2, and TGFβ signaling pathways between cell pairs with significant interactions. The color and size of the dots in the scatterplot represent the range of *P*-value and the possibility of cellular communication, respectively. (J) GO enrichment BarPlot and CircularPlot of differentially expressed genes with and without TGFβ-treated kidney organoids derived from two independent young donors (34y, 41y). Enrichment was shown with the percentage of genes (left) or *P*-value (right) with details in Table S1. (K) The relative cell viability of TNFα-treated organoids with or without TGF-β (MCE, HY-P7118, 500 ng/mL, 48 h) application. (L) Schematic representation of pathway characteristics of kidney organoids upon TNFα treatment in young and old donors. Data are presented as mean ± SEM and *, *P* < 0.05; **, *P* < 0.01; ***, *P* < 0.001; ****, *P* < 0.0001 compared with a control group. *n* = 6 per group for bulk RNA-seq data and *n* = 1 per group for scRNA-seq data.

Unexpectedly, a series of TNFα-induced downstream pro-fibrotic responses which were enriched in TGFβ pathways including TGFβ2, TGFβ3, BMP3, and LEFTY1, were found to be significantly down-regulated in the younger group. Given the critical role of TGFβ in driving renal fibrosis, it is imperative to investigate whether the reduction of its associated pathway following a short-term pulse of TNFα has a protective effect against injury. To facilitate the interpretation of intercellular communication networks, CellChat was utilized to quantitively measure networks through different clusters of TNFα treated organoids. The results revealed that ILC possessed multiple sources of ligands targeting IRC cells (Fig. 2G). TNFα interacted as the driver initiation factor in EC, ILC, and IRC cell clusters. Besides, “GDF–TGFβ” and “BMP–BMPR” signaling pathways had similar cell–cell talk patterns, representing the involvement of TGFβ signals in TNFα-stimulated response (Fig. 2H). The bubble chart results of the interactions between key pathways also showed that TGFβ signaling frequently interacts among EC, ILC, and IRC subpopulations upon TNFα stimulation (Fig. 2I).

To further investigate the role of TGFβ on renal organoids, we then treated organoids with TGFβ to mimic profibrotic response and injury. Bulk RNA-seq showed a significant reduction of cell cycle-related pathways and DNA replication by GO analysis (Fig. 2J, Table S1), suggesting a distinct mechanism of injury response induced by TGFβ compared to TNFα induction. Indeed, the cell viability assay confirmed a significant reduction in kidney organoid growth (Fig. 2K). Our results revealed an enhanced inflammatory response of kidney organoids from young donors, which may be partially attributed to the downregulation of TGFβ-associated pathways (Fig. 2L).

In summary, we have established an epithelial inflammatory injury model using adult kidney organoids to provide novel tools for studying inflammation-related kidney disease and facilitating drug discovery.

## Research limitations

Our study illustrated that ASC-derived kidney organoids can recapitulate the inflammatory response following TNFα treatment. However, future work involving immune cells, vessels, and interstitial cells would be useful to better understand their role in modeling inflammation-related nephropathy with the technical support of organ-on-a-chip or co-culture methods. In addition, further mechanistic studies on the crosstalk of TNFα and TNFβ signaling pathways with age are required to guide potential clinical treatments targeting inflammatory networks. Meanwhile, the precise reason for CD69 activation in kidney organoids remains unclear.

## Supplementary Material

lnae022_suppl_Supplementary_Materials
