# Supplementary material for: Establishment of epithelial inflammatory injury model using adult kidney organoids
Source: Life Med. 2024 May 9;3(3):lnae022. doi: 10.1093/lifemedi/lnae022 (PMC11749467; doi:10.1093/lifemedi/lnae022)
Supplement: lnae022_suppl_Supplementary_Materials [file lnae022_suppl_Supplementary_Materials.docx]

**Methods**

**Tissues**

All kidney tissues were derived with the permission of the policy approved by the Medical Ethics Committee of Qilu Hospital of Shandong University (NO. KYLL-202204-069) and comply with all relevant ethical regulations. Written informed consent was obtained from the patients or donors. Biopsies were isolated and transported on ice at 4°C in DMEM/F12 supplemented with 1×penicillin/streptomycin, 1×HEPES(Invitrogen), 1×Glutamax (Invitrogen).

**Organoid culture and** **treatment with TNF-α and TGF-β**

Tissues derived from kidney biopsy or kidney donors were minced, digested with collagenase for 45 min at 37°C. Digested single cells were passed through a 70 μm filter, and then planted in Matrigel (Corning) or Basement Membrane Extract (BME, R&D Systems) with culture medium. The culture medium included: Advanced DMEM/F12 supplemented with 1×penicillin/streptomycin, 1×HEPES (Invitrogen), 1×Glutamax (Invitrogen), 1×B27 (Invitrogen), and N-acetylcysteine (1mmol/l, Sigma-Aldrich), EGF (50 ng/ml, Peprotech), Rho-kinase inhibitor Y-27632 (10 μM, selleck), A83-01 (5 μM, Tocris Bioscience) and Primocin (0.1 mg ml-1, Invivogen) and Recombinant Human R-Spondin-1(100 ng/ml, Peprotech). Organoids were treated with 100ng/ml, 250ng/ml, 500ng/ml, 1000ng/ml TNF-α (Origene, TP750007-1000) for 24 hours, 48 hours or 120 hours, respectively. For TGF-β treatment, organoids were treated with 500ng/ml TGF-β (MCE, HY-P7118) for 48 hours.

**Whole mount immunofluorescence**

Organoids were thoroughly isolated from Matrigel or BME with the addition of Cell Recovery Solution (Corning) on ice for 30 minutes. Organoids were fixed in 4% (wt/vol) paraformaldehyde in PBS for 45 min, washed with 0.1% Triton X-100 and 0.2% BSA in PBS (OWB) twice. Organoids were incubated with 200 μl OWB including primary antibodies (2×concentration) to each well overnight at 4 °C with mild shaking. The organoids were washed with OWB three times and incubated with 200 μl of OWB with secondary antibodies (2×concentration) per well with mild shaking for 2 hours or overnight. Antibodies were washed and then fructose-glycerol clear solution was used by pipetting using a 200 μl pipette tip with clipped ends. Immunofluorescence images were acquired using a confocal microscope (Zeiss LSM880). Images were analyzed and processed using the software ZEN 3.4.

**Cell viability Assays**

Organoids were digested into single cells and seeded into 96-well plates with the concentration of 3500 cells per well. The CellTiter-Glo® 3D Reagent (Promega, USA) was added to each well of 96-well plates. Cell lysis was then induced by vigorously pipetting the contents for 5 min, and the plate was incubated at 37°C for 25 min to stabilize the luminescent signal (light free). Then, 75 µl of the supernatant were transferred in technical replicates to 96-well opaque wall plates for luminescent measurement.

**RNA isolation and qRT-PCR analysis**

The RNA was isolated with Trizol reagents (Invitrogen) according to the manufacturer’s instruction. RNA was reversely transcribed into cDNA by reverse transcriptase (Vazyme, Nanjing, China). Quantitative PCR analysis was performed using gene-specific primers listed in Supplementary Table S2 and HiScripter Ⅱ QRT SuperMix for qPCR (Vazyme, Nanjing, China).

**Bulk RNA sequencing and data analysis**

Organoids were collected for transcriptional analyses for different experimental conditions. The RNeasy Micro Kit (Qiagen, 74004) was used for RNA extraction following the manufacturer’s protocol.

A total amount of 1 µg RNA per sample was used as input material for the RNA sample preparations. Sequencing libraries were generated using NEBNext® UltraTM RNA Library Prep Kit for Illumina® (NEB, USA) following manufacturer’s recommendations and index codes were added to attribute sequences to each sample. Quantitative polymerase chain reaction (qPCR) was performed using the Taqman Fast Advanced Master Mix (Thermo Fisher, 444557) following the manufacturer’s instruction on the ViiA 7 Real-Time PCR System (Thermo Fisher). Bulk RNA sequencing data in our manuscripts are available in GSE259376. Sequencing results were mapped using the DESeq2 R package (1.16.1) and differential expression analysis of comparative groups was performed. A corrected P-value of 0.05 and the absolute fold change of the two were set as thresholds for significant differential expression. Bioinformatic analysis was performed using Software R or the OmicStudio tools at <https://www.omicstudio.cn/tool>.

**Single-cell RNA-seq of kidney organoids**

Kidney organoids were harvested and dissociated into single cells using Trypsin-EDTA (Gibico). Single-cell suspensions (2×10^5^ cells/ml) with PBS (HyClone) were loaded onto microwell chip using the Singleron Matrix® Single Cell Processing System. Barcoding Beads were subsequently collected from the microwell chip, followed by reverse transcription of the mRNA captured by the Barcoding Beads and to obtain cDNA, and PCR amplification. The amplified cDNA was then fragmented and ligated with sequencing adapters. The scRNA-seq libraries were constructed according to the protocol of the GEXSCOPE® Single Cell RNA Library Kits (Singleron). Individual libraries were diluted to 4 nM, pooled, and sequenced on Illumina novaseq 6000 with 150 bp paired end reads. ScRNA-seq data were deposited in the Gene Expression Omnibus (NCBI) under accession number GSE259381.

**Organoid ELISA**

Protein levels in kidney organoid culture media and lysates were measured using commercially available ELISA kits for CXCL5 (proteintech, KE00271), CXCL8 (DRKEWE, 1110802), and CCL20 (proteintech, KE00149). Samples were processed in duplicate following the manufacturer’s protocol. Values were normalized to total protein content using Pierce BCA protein assay (Thermo Scientific, cat#23227). At least two independent experiments were performed and plotted using GraphPad Prism software. Statistical significance was calculated using unpaired Student’s t test.

**Statistical analysis**

Statistical analyses were conducted with Prism software (GraphPad, La Jolla, CA). For two independent group comparison two-tailed t-test was used. Data are presented as mean ± SEM and P values determined by Student t test; *p <0.05. **p <0.01. ***p <0.001. ****p <0.0001 was considered statistically significant.

**Table S1 The supplement of Fig.2J**

| GO_ID | GO_Term | GO_Category | P.value |
| --- | --- | --- | --- |
| GO:0000732 | strand displacement | BP | 8.49E-08 |
| GO:0071897 | DNA biosynthetic process | BP | 3.32E-06 |
| GO:0000075 | cell cycle checkpoint | BP | 5.28E-06 |
| GO:0000731 | DNA synthesis involved in DNA repair | BP | 1.62E-05 |
| GO:0006260 | DNA replication | BP | 1.89E-05 |
| GO:0007095 | mitotic G2 DNA damage checkpoint | BP | 1.98E-05 |
| GO:0044818 | mitotic G2/M transition checkpoint | BP | 0.000035 |
| GO:0031572 | G2 DNA damage checkpoint | BP | 3.53E-05 |
| GO:0007059 | chromosome segregation | BP | 5.86E-05 |
| GO:0001325 | formation of extrachromosomal circular DNA | BP | 7.71E-05 |
| GO:0090656 | t-circle formation | BP | 7.71E-05 |
| GO:0090737 | telomere maintenance via telomere trimming | BP | 7.71E-05 |
| GO:0007093 | mitotic cell cycle checkpoint | BP | 7.89E-05 |
| GO:0060337 | type I interferon signaling pathway | BP | 0.000094 |
| GO:0071357 | cellular response to type I interferon | BP | 0.000094 |
| GO:1903046 | meiotic cell cycle process | BP | 0.000137 |
| GO:0034340 | response to type I interferon | BP | 0.000193 |
| GO:0051321 | meiotic cell cycle | BP | 0.000231 |
| GO:0000280 | nuclear division | BP | 0.000233 |
| GO:0032647 | regulation of interferon-alpha production | BP | 0.000239 |
| GO:0098687 | chromosomal region | CC | 4.69E-05 |
| GO:0005871 | kinesin complex | CC | 0.000127 |
| GO:0004520 | endodeoxyribonuclease activity | MF | 8.13E-06 |
| GO:0140097 | "catalytic activity, acting on DNA" | MF | 4.94E-05 |
| GO:0003725 | double-stranded RNA binding | MF | 5.23E-05 |
| GO:0004536 | deoxyribonuclease activity | MF | 5.23E-05 |
| GO:0008094 | DNA-dependent ATPase activity | MF | 6.27E-05 |
| GO:0016887 | ATPase activity | MF | 0.000278 |

**Table S2 The supplement of Fig.1A**

| Primer name | Sequence |
| --- | --- |
| CXCL8-R | CAACAGACCCACACAATACATGA |
| CXCL8-F | GAAGTTTTTGAAGAGGGCTGAGA |
| IL-6-R | GGGCGGCTACATCTTTGGAA |
| IL-6-F | CTCCTTCTCCACAAGCGCC |
| IL-1β-R | GTCCTGGAAGGAGCACTTCAT |
| IL-1β-F | AACCTCTTCGAGGCACAAGG |
| TNF-α-R | TGAGGTACAGGCCCTCTGAT |
| TNF-α-F | GCCCATGTTGTAGCAAACCC |
